# An *in vitro* method to manipulate the direction and functional strength between neural populations

**DOI:** 10.3389/fncir.2015.00032

**Published:** 2015-07-14

**Authors:** Liangbin Pan, Sankaraleengam Alagapan, Eric Franca, Stathis S. Leondopulos, Thomas B. DeMarse, Gregory J. Brewer, Bruce C. Wheeler

**Affiliations:** ^1^J. Crayton Pruitt Family Department of Biomedical Engineering, University of FloridaGainesville, FL, USA; ^2^Department of Biomedical Engineering, University of California IrvineIrvine, CA, USA

**Keywords:** MEMS (Micro Electro Mechanical Systems), functional connectivity, structure-activity relationship, multielectrode array, dissociated neuronal culture, cortical synchronization, *in vitro*

## Abstract

We report the design and application of a Micro Electro Mechanical Systems (MEMs) device that permits investigators to create arbitrary network topologies. With this device investigators can manipulate the degree of functional connectivity among distinct neural populations by systematically altering their geometric connectivity *in vitro*. Each polydimethylsilxane (PDMS) device was cast from molds and consisted of two wells each containing a small neural population of dissociated rat cortical neurons. Wells were separated by a series of parallel micrometer scale tunnels that permitted passage of axonal processes but not somata; with the device placed over an 8 × 8 microelectrode array, action potentials from somata in wells and axons in microtunnels can be recorded and stimulated. In our earlier report we showed that a one week delay in plating of neurons from one well to the other led to a filling and blocking of the microtunnels by axons from the older well resulting in strong directionality (older to younger) of both axon action potentials in tunnels and longer duration and more slowly propagating bursts of action potentials between wells. Here we show that changing the number of tunnels, and hence the number of axons, connecting the two wells leads to changes in connectivity and propagation of bursting activity. More specifically, the greater the number of tunnels the stronger the connectivity, the greater the probability of bursting propagating between wells, and shorter peak-to-peak delays between bursts and time to first spike measured in the opposing well. We estimate that a minimum of 100 axons are needed to reliably initiate a burst in the opposing well. This device provides a tool for researchers interested in understanding network dynamics who will profit from having the ability to design both the degree and directionality connectivity among multiple small neural populations.

## Introduction

In living neural networks the relationship between a network’s structural connectivity and its functional properties are influenced by a number of variables. Perhaps one of the most well known of these are the connection strengths that bind individual neurons and at larger spatial scales, the number and size of fibers of passage that influence the strength of communication between brain areas (Boussaoud et al., 1990; Felleman and Van Essen, 1991; Kaas and Collins, 2001; Markov et al., 2011). The importance of this topic is highlighted by a recent review (Feldt et al., 2011) and the US and European initiatives in brain mapping. Quantification of connection strength is perhaps one of the most fundamental steps towards a better understanding of a brain network’s function (Olson and Musil, 1992; MacNeil et al., 1997; Scannell et al., 2000; Markov et al., 2011). In this study we present a method based on Micro electro mechanical systems (MEMs) technology and approach using this technology to manipulate and then evaluate the effect of varying connection strengths between small neural populations *in vitro*.

Although microtunnel-like devices have an extensive history (Campenot, 1977), it is only recently that this technology has become popular in neuroscience research within dissociated (Taylor et al., 2003, 2005, 2009; Pearce et al., 2005; Berdondini et al., 2006; Morin et al., 2006; Ravula et al., 2007; Feinerman et al., 2008; Liu et al., 2008a; Dworak and Wheeler, 2009; Park et al., 2009; Yang et al., 2009; Berdichevsky et al., 2010; Shi et al., 2010; Taylor and Jeon, 2010; Wieringa et al., 2010; Kanagasabapathi et al., 2011, 2012, 2013; Pan et al., 2011; Peyrin et al., 2011; Biffi et al., 2012; Bisio et al., 2014; Sung et al., 2014; Tang-Schomer et al., 2014) and organotypic culture (Berdichevsky et al., 2010, 2012). *In vitro* neuronal cell-culture preparations used in combination with this technology may provide a promising new way to directly manipulate and study the effects of connection strength upon network activity under ideal conditions due to their accessibility and flexibility (Maeda et al., 1995; Potter, 2001; Pan et al., 2009b; Levy et al., 2012). These devices, when combined with multielectrode array (MEA) technology for simultaneous electrophysiology (Dworak and Wheeler, 2009), provide the means to directly manipulate the structural properties of networks while simultaneously monitoring its effect on a network’s functional dynamics.

**Figure 1:**
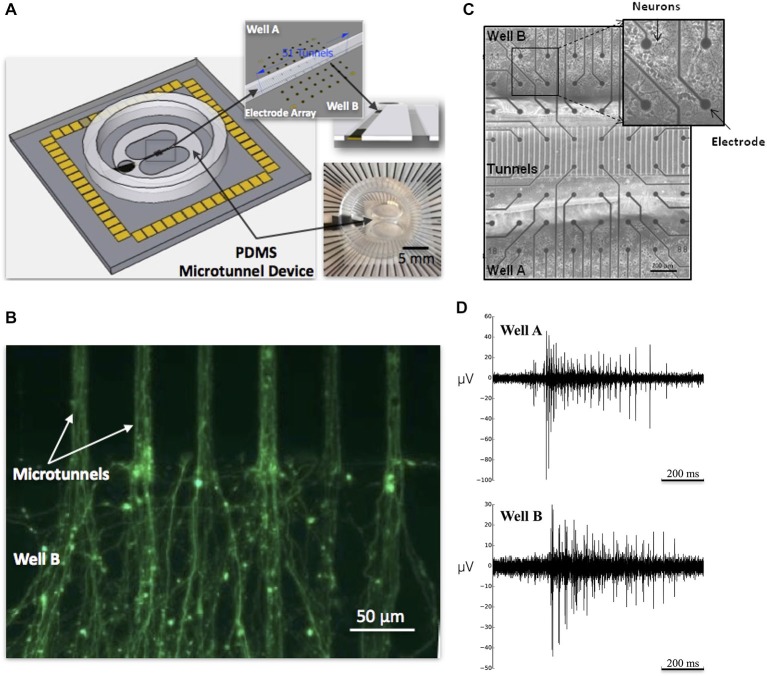
**Microtunnel device and neuronal culture of cortical neurons. (A)** The large schematic illustrates a full view of a microtunnel device attached on an multielectrode array (MEA). A picture of a device with MEA is shown in the lower right inset; the upper right inset depicts the alignment of the microtunnels with the electrode array. **(B)** Calcein stained axons growing through microtunnels from neurons in placed in Well A and extending into Well B. At this point neurons would be added to Well B to establish feed-forward connectivity between small living neuronal networks in Well A and B. **(C)** Micrograph of 8 × 8 grid of electrodes and 51 tunnel microtunnel device plated with cortical neurons in Well A and Well B. **(D)** Raw electrophysiology recorded from a single electrode in Well A and one in Well B from a single MEA in the 10 tunnel group depicting action potentials recorded during a burst event that propagated between wells.

The device, illustrated in Figure 1A, is composed of two wells, labeled Well A and Well B, each containing a small population of cultured neurons (Figure 1C) that are interconnected by micro-scale tunnels (Figures 1A,B). Each device is positioned and attached to the surface of an MEA to permit recordings in Well A, Well B, and select tunnels that connect each well (Figure 1A; upper right). By staggering the times at which cells are placed into the first well and then later the second, our laboratory can now create cultured networks in which two small neural populations can be connected with predominantly unidirectional connectivity from Well A to B or *vice versa*, as measured by delays of both individual action potentials detected along axons and bursts of activity within communicating populations of neurons (Pan et al., 2011, 2014; Bisio et al., 2014). With these devices we show that by manipulating the number of tunnels that lie between each well from 2 to 5, 10, 15 and 51 we can affect the overall strength of functional connectivity connecting each neural population, influence the probability of successful transmission of bursts of activity from one well to another, and characterize the changes in dynamics produced by differing number of tunnels.

## Materials and Methods

### Microtunnel Device Fabrication

**Figure 2:**
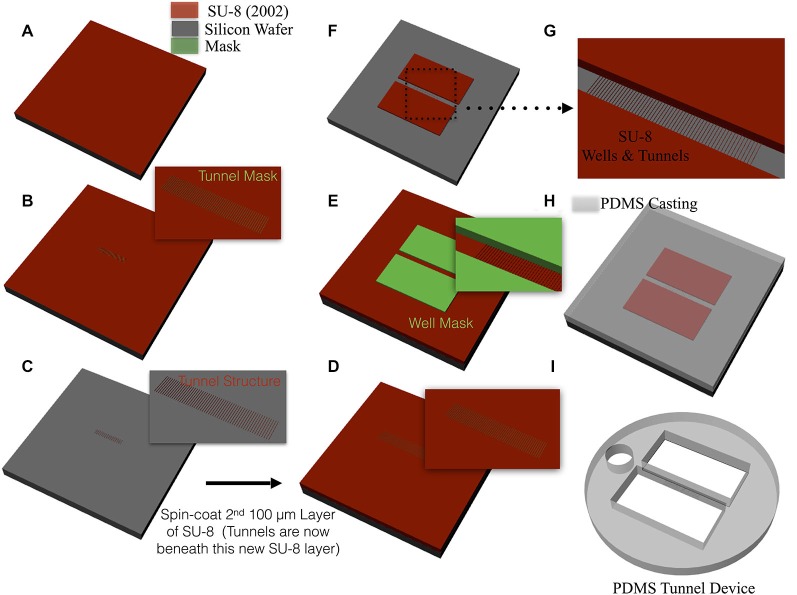
**Mold fabrication and device casting. (A)** A thin 3 μm layer of SU-8 was spin-coated onto the surface of a 4 inch silicon wafer. **(B)** A mask containing the tunnel pattern is placed over the SU-8 and then exposed to UV, baked, and developed to create the tunnel structure shown in **(C)**. SU-8 was again spin coated upon the surface to a final thickness of 120 μm **(D)** and a second mask was overlaid that contained the well pattern **(E)**. The surface was again exposed to UV, baked, and developed to create the negative of the well and tunnel features **(F,G)** that will later be used for casting the device. **(H)** PDMS is slowly poured onto the surface the wafer, allowed to cure in a 95°C oven for two hours, and any extraneous PDMS material removed with biopsy punches to produce the final device in **(I)**. Each device was then aligned and attached over a grid of MEA electrodes that recorded neural activity.

Each device, illustrated in Figure 1A, was constructed of polydimethylsilxane (PDMS) and consisted of two 3 × 10 mm wells separated by a central 400 μm bridge of material containing the tunnels. Each device was manufactured by casting from a mold fabricated using photolithographic techniques illustrated in Figure 2. To create the microtunnel area of the mold, SU-8 2002 (Microchem Inc., Santa Clara, CA, USA) was spun onto a 4-inch silicon wafer at a nominal thickness of 3 μm, baked at 95°C for 3 min, exposed with the first mask, baked at 95°C again for 5 min and developed in SU-8 developer for 20 s. The developed image was sprayed and washed with fresh developer for 10 s, followed by a second spray/wash with Isopropyl Alcohol (IPA) for another 10 s before it being air dried with filtered, pressurized nitrogen. The second part of the mold, which defines the well structure, was made using SU-8 2050 (Microchem, Inc.), which was spun onto the surface at a nominal thickness of 120 μm and then baked at 95°C for 30 min. The second mask was aligned with the alignment marks of the first SU-8 film and then the second SU-8 film was exposed, baked again at 95°C for 15 min and developed in SU-8 developer for 9 min. The developed image was sprayed and washed with fresh developer for 10 s, followed by a second spray/wash with IPA for another 10 s before it was air dried with filtered, pressurized nitrogen. At this point the mold was ready for casting the PDMS microtunnel devices. PDMS (Monomer: Curing agent w/w ratio was 10:1, Dow Corning Corporation, Midland, MI, USA) was poured onto the wafer slowly and allowed to spread over the entire surface. Each was placed on a hotplate at 70°C during the two-hours required for curing. The layer of cured PDMS was then peeled off the wafer. Two wells for the culture and a third smaller 2 mm circular hole for the reference electrode were formed using commercial biopsy punches. Final dimensions of each microtunnel were 3 μm tall, 10 μm wide, 400 μm long, and spaced 40 μm apart (center-to-center). Devices were manufactured with 2, 5, 10, 15 or 51 tunnels. A more detailed description of the fabrication of these devices can be found in our earlier paper (Pan et al., 2011).

### Cell Culture

The surface of each MEA was coated overnight with poly-D-lysine (PDL) solution (100 μg/ml, diluted in borate buffer at pH of 8.5) prior to cell culture to promote cell adhesion and growth in a monolayer. Each MEA (60MEA200/30iR-Ti-w/o, Multi Channel Systems, Inc., Reutlingen, Germany) consisted of 59 TiN electrodes (30 μm in diameter) arranged in an 8 × 8 grid spaced 200 μm apart as shown in Figure 1A. On the following day each MEA was rinsed three times with sterilized DI water and then dried. Each microtunnel device was aligned with the 8 × 8 grid of electrodes and attached using a customized aligner (XYZ plus three angular rotations) such that two of the eight rows of the 8 × 8 grid of electrodes were located beneath the microtunnels and three rows of electrodes were located within each well as shown in Figure 1A. Each MEA with attached device was initially filled with Neurobasal^TM^/B27/GlutaMAX^TM^ (Invitrogen, Inc.) media and incubated at 5% CO_2_ and 37°C for several hours to ensure that the media would penetrate into the tunnels before any cells were plated. The media was then aspirated from the wells and was then ready for cell plating.

**Figure 3:**
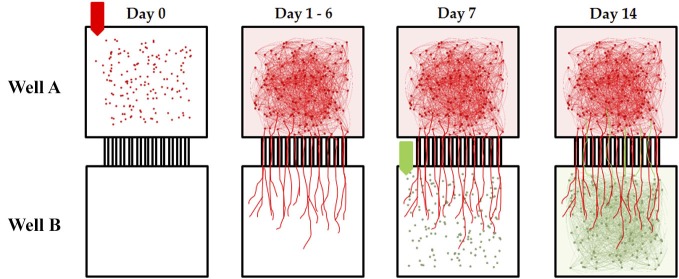
**Timed sequential plating of cortical neurons for feed-forward connectivity.** Each micro-tunnel device was aligned and attached to a 60 electrode MEA. Cortical neurons were first seeded in Well A on Day 0. During the following 6 days neurites extend from soma in Well A and synapse with other soma in Well A while others enter the tunnels and extend into the adjacent well. Eventually enough axons have entered the tunnels that on Day 7 when additional neurons are added to Well B there is little room for neurites from those new neurons to cross back into Well A. The result is a primarily feed-forward network in which the majority of cross-well connectivity is biased in one direction (Well A to Well B). In this paper we manipulate the number of tunnels that connect each chamber and measure the effect this has on the “strength” of the connection between two neural populations separated by these tunnels.

Embryonic E18 rat cortical hemispheres purchased from BrainBits LLC (Springfield, Illinois, USA) were dissociated according to the vendor’s protocol. An MEA with an attached microtunnel device was removed from the incubator and the media aspirated from the first well, which we will refer to as Well A. Twenty μl of cell suspension (3,000,000 cells/ml) was then added to well A (Figure 3, Day 0). Each MEA was then placed in the incubator for 10 min to permit cells to attach to the surface. Then 300 μl Neurobasal^TM^/B27/GlutaMAX^TM^ media was added into the media chamber of each device, providing a reservoir large enough to withstand evaporation losses. We used serum free Neurobasal^TM^/B27/GlutaMAX^TM^ media originally formulated to maximize neural survival at the expense of glial growth at 4 days *in vitro* (Brewer et al., 1993; Brewer, 1995). However, after two weeks in culture glial populations recover to a density approximately that of normal culturing conditions (Nam et al., 2004, 2007). Each MEA with attached device was then incubated at 5% CO_2_ and 37°C. Half of the media was changed every 2 days. During this time neurites gradually extend from soma in Well A into the tunnels (Figure 3, Day 1–6) reaching the opposite chamber and eventually filling the tunnels in about 5–7 days (Figure 3, Day 1–6). At day 7, the media was removed from Well B only, and very quickly, cells were plated in Well B with the same density as Well A (Figure 3, Day 7). Ten minutes later 300 μl of media was added into each media chamber and the MEA was then returned to the incubator. Because the tunnels are blocked by neurites from Well A, the majority of neurites from Soma in Well B remain within Well B and establishing a primarily feed forward structure from Well A and Well B (Pan et al., 2011, 2014; Figure 3, Day 14). The ages of the cultures (days *in vitro* or DIV) in this report are all referred to the date of the initial plating in well A. Recordings were conducted during DIV 21 to 28 at 37°C and 5% CO_2_ (balance air). The cell harvesting procedure was approved by the University of Florida and SIUSM animal care committees.

### Cell Staining

An additional set of three microtunnel devices were attached to glass coverslips (without electrodes) and cultured with neurons in a single well for 6 days to perform fluorescent cell staining to image axonal growth. After six days each culture was rinsed with PBS twice before 2 μM Calcein-AM (Invitrogen) in DMSO was added. Cells were then incubated for 1 h at room temperature before examination of the tunnel area under a microscope with a fluorescein optical filter (485 nm). Staining was not performed directly on the MEAs to avoid damage and or toxicity during subsequent reuse.

### Data Acquisition and Analysis

Neuronal activity was recorded using a commercial multichannel signal amplifier (MEA 1060BC, Multi Channel Systems, Inc., Reutlingen, Germany) with a gain of 1200. Signals were sampled at a rate of 25 kHz and controlled by the data acquisition software provided by Multi Channel Systems (MCRack v3.9.1, Multi Channel Systems, Inc., Reutlingen, Germany). Action potentials were detected in the extracellular electrophysiological recordings provided by the 8 × 8 grid of MEA electrodes using a negative threshold of 5 times the standard deviation estimated from background noise (during periods of relative inactivity). Figure 1D shows examples of raw electrophysiology from two of the 59 recording electrodes depicting a burst of action potentials. Mean firing rates (spike rates) were calculated as the mean firing rate across electrodes and mean across MEAs to represent firing rates for each group. Network bursts were detected separately within each layer using the summex method (Wagenaar et al., 2005). Briefly, spike trains for each electrode within a layer were searched individually for burstlets (sequences of at least four spikes with interspike intervals less than a threshold set to 25% of that electrodes inverse average spike detection rate). Any group of burstlets across channels that overlapped in time was considered a burst. Minimum burst durations were enforced at 10 ms.

To estimate functional connectivity among neurons within each network or between the two networks (i.e., between neurons separated by tunnels) a single electrode in Well A was electrically stimulated. Each stimulus would evoke a burst of activity among neurons in this well which could potentially propagate via axons through the tunnels into the network neurons in Well B. Electrical stimulation was provided by a commercial stimulus generator (STG 2004, Multi Channel Systems, Inc., Reutlingen, Germany). Although the blanking circuit present in the amplifier enabled some suppression of electrical stimulation artifacts during recording, we enforced a 5 ms blanking period from the stimulus onset and removed the stimulated channel from analysis to ensure recordings were not corrupted. Any remaining electrical artifacts were removed offline using a locally fitted polynomial (Wagenaar and Potter, 2002). An electrode in Well A was selected along the outside row of electrodes furthest from the tunnels such that a probe stimulus (±800 mV/200 μs per phase) applied to that electrode reliably evoked a burst of activity across the population of neurons within that well. Probes were then repeated every 10–15 s for a maximum of 60 stimuli in each culture.

During each trial the electrical stimulus in Well A would evoke a population wide burst of neural activity in Well A that would sometimes propagate into Well B. However, the likelihood and delay between an evoked burst in Well A and burst propagated by neural activity into Well B appeared dependent on the number of tunnels. To quantify the likelihood of successful propagation we computed the percentage of bursts evoked by a stimulus in Well A that successfully propagated into Well B. We also estimated the delay between the time the stimulus was applied and a burst appeared in the opposing well in two ways: peak-to-peak and time to first spike.

#### Peak-To-Peak Propagation Delays

**Figure 4:**
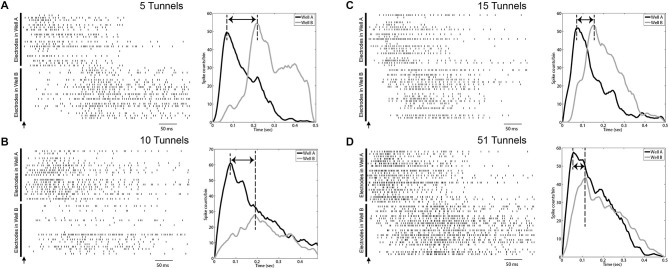
**Evoked burst activity propagates across wells with dependence on number of connecting tunnels.** Left: Examples of raster plots of evoked bursts recorded in both wells interconnected by 5 **(A)**, 10 **(B)**, 15 **(C)** and 51 microtunnels **(D)**. The arrow shows the time when the stimulus pulse was applied on a corner electrode in Well A. Each row represents a spike train recorded by a single electrode. The upper portion of each raster indicates electrodes in Well A and the lower part represents electrodes in Well B. The right most portion of each panel also depicts a post-stimulus time histogram (PSTH, 20 ms bins) of spiking summed over electrodes in Well A (black line) and Well B (gray line) associated with the raster in each panel. Time zero indicates the time of the stimulus pulse. The electrical artifacts from stimulation are not included. All graphs were smoothed with a Gaussian filter whose width was three bins. Delay times shown are from the peak in Well A to the peak in Well B.

First we constructed a post stimulus time histogram (PSTHs, bin widths of 5 ms) for each burst event from the spike data from each electrode in the stimulated and opposing well during a 500 ms window following each stimulus. The PSTHs for individual electrodes were then averaged to obtain a mean PSTH representing the temporal profile that was then used to assess propagation delays between each well. Peaks were detected within each PSTH in Well A and again in Well B and time between peaks from Well A to Well B tabulated (Eytan and Marom, 2006). This difference in time represented the delay that we observed between the network-wide burst of neural activity evoked immediately in Well A and the latency with which that burst was able to propagate across the tunnels to initiate a burst in Well B (See Figure 4).

#### Time to First Spike

Another common approach to timestamp burst events is based on the spike times of the first few spikes that occur during each event. For example, information derived from first spikes have been used to estimate propagation velocities (Maeda et al., 1995), and used to help identify putative “leader” neurons or “initiation zones” among bursts within small cultured neural populations like those used here (Yvon et al., 2005; Eytan and Marom, 2006; Eckmann et al., 2007, 2008; Cohen et al., 2008; Ham et al., 2008; Pan et al., 2009a; Orlandi et al., 2013). In our prior report, we based our estimates of the directionality based on the timing of the spikes that appeared along electrodes located along the tunnels separating each well to determine which direction spikes were traveling and confirmi feed-forward propagation from Well A to Well B (Pan et al., 2011). Unfortunately the simple form of this metric was unreliable due to the evoked nature of our data. In this case each stimulus tended to evoke a direct response (a few action potentials) from electrically activated neurons in Well A and a handful of spikes, presumably some due to ongoing spontaneous activity, in Well B that were not associated with the main body of the burst in Well B. This problem is apparent in the raster plot in Figure 4A in which a stimulus delivered to a single electrode in Well A rapidly produced a network wide burst of activity in Well A as expected. However, in Well B the high rate of firing during the early phase of that activity in Well A translated into sporadic firing in Well B, but with the main body of the burst delayed over 100 ms following the stimulus. In this case a simple first spike measure would fail to capture the delay between these two burst events.

In response, we modified the detection procedure for first spikes to search within the strongest portion of the neural response in Well B. In this study, we operationally define a “first spike” as that spike which first appeared in our recordings following the crossing at progressively higher thresholds of 10, 20, or 50% prior to peak firing rates (i.e., PSTH height) was calculated during each evoked response. Delays were represented by the time from a first spike in Well A to a first spike in Well B estimated at each threshold. Each successively higher threshold partially eliminates the problem of including spikes that are not associated with the principal burst event in Well B. We report the average of these delays between first spikes for each stimulated burst event.

### Functional Connectivity Analysis

A variety of methods are now available to measure the functional connectivity among neurons within a neuronal population. A traditional approach would compute the cross correlation among spike trains recorded on each electrode (Gerstein and Perkel, 1969; Aertsen and Gerstein, 1985; Aertsen et al., 1989). However this approach is widely known to be susceptible to nonstationarities in firing rates such as those in the presence of bursting which is the predominant network response to stimulation in these cultures. A number of alternative methods have now become popular to estimate functional connectivity including transfer entropy (Stetter et al., 2012), directed transfer function (Kamiński and Blinowska, 1991; Eichler, 2006), partial directed coherence (Sameshima and Baccalá, 1999; Takahashi et al., 2007), Granger Causality (Fanselow et al., 2001; Ding et al., 2006; Cadotte et al., 2008; Kispersky et al., 2011), measures from information theory (Borst and Theunissen, 1999; Rieke et al., 1999; Dayan and Abbott, 2005), and direct estimation of structural morphology (e.g., Ullo et al., 2015). There are now a number of examples of functional connectivity measures have been used to delineate connectivity from spike trains recorded with MEAs (e.g., Bettencourt et al., 2007; Garofalo et al., 2009; Feldt et al., 2010; Kanagasabapathi et al., 2011; Downes et al., 2012; Maccione et al., 2012; Pirino et al., 2015) and data from tunnels devices similar to those used here (Kanagasabapathi et al., 2012). In this paper we computed a conditional form of Granger causality (CGC; Kamiński and Blinowska, 1991) based on our work adapting CGC to spike trains measured using *in vitro* MEA technology (Cadotte et al., 2008) and reports by other laboratories (Kispersky et al., 2011). Unlike traditional pair-wise Granger causal comparisons, CGC enables estimation of the pairwise Granger causal strength of connections between neurons (electrodes) conditioned on the strength of any potential intermediate nodes that may also causally contribute to activity within this pair.

In simplistic form, Granger Causality begins by computing the best linear predictive filter (for the next sample) based on past samples of a signal; then a comparison is done between the improvement in prediction gained by including samples from a second signal, permitting quantification of the enhancement of the prediction and with imputation of degree of causality to the degree of improvement of the prediction. When comparing pairs of signals, the signal whose inclusion most improves the prediction of the other is presumed to be most causal. Tests of significance exist to distinguish putatively causative influence from incidental correlation.

Here, CGC was estimated between all pairs of electrodes (both under the tunnels and wells) using GCCA toolbox developed by Seth (2010) based on the method we developed earlier for smoothed spike trains constructed from the stimulus evoked data measured using MEAS (Cadotte et al., 2008). Briefly, action potentials were detected in a window 500 ms following each stimulus. Spike times were binned at intervals of 1 ms and smoothed using an exponentially decaying waveform with time constant of 4 ms to produce a continuous wave-form suitable for CGC analysis. The Granger causal strength between each pairwise comparison between electrodes was then computed using CGC. Only electrodes for which the spike rate exceeded 0.5 Hz were included in the analysis. CGC values were calculated by fitting the smoothed waveform to a multivariate autoregressive process of order 10 and obtaining the ratio of the residuals. Each value was determined to be statistically significant if the corresponding coefficients of the multivariate auto regressive process were jointly significantly different from zero. The threshold test was then corrected with for the false discovery rate to account for multiple comparisons (*p* < 0.001; Seth, 2010). Any Granger causal estimates between electrode pairs that were above this statistical threshold were included in any subsequent analysis.

Graphical representations of each network’s functional connectivity were created using Gephi (Bastian et al., 2009) based on significant pairwise CGC estimates. In this representation, the nodes increase in proximity (i.e., draw together) as the Granger-causal estimate of functional strength between those nodes increases. The thickness of the connecting lines denotes the Granger-causal strength between those nodes. (See also Figure 6A).

Statistical analysis was performed using Matlab and consisted of one-way ANOVA with group as the factor. *p* < 0.05 were considered to be significant. Error bars in graphs denote the 95% confidence interval of mean. There were four MEA cultures in the 2 and 51 tunnel groups, five in the 5 and 15 tunnel groups, and six in the 10 tunnel group.

## Results

### Neurite Connectivity

Neurites were observed entering the microtunnels from Well A as early as the first day following plating (i.e., DIV 1). On DIV 6, axons from Well A appeared to occupy the majority of the volume within each microtunnel and now extended more than 200 μm into Well B. Based on the fluorescent images (Figure 1B), the average number of axons that emerged from a single microtunnel on DIV 6 was visually estimated at 10.3 ± 5.7 (*n* = 34 microtunnels). Prior reports have shown that almost all the traversing neurites within tunnels whose structure is longer than 400 μm are axons (Taylor et al., 2005, 2009).

### Effect of Functional Strength on Burst Propagation, Delays, and Success Rates

After two weeks in culture spontaneous network wide bursts of neural activity could be observed during recordings using the MEA electrodes located beneath the two wells and communication between wells observed from activity measured within the tunnels. Mean firing rates at each electrode did not differ significantly for the 2, 5, 10, 15 and 51 tunnel groups (0.94 ± 0.24, 1.43 ± 1.13, 1.29 ± 0.81, 2.32 ± 2.35, 1.6 ± 0.82 spikes per sec, respectively, *p* > 0.16). There were also no significant differences (*p* > 0.21) in the rate of bursting between groups with 0.71 ± 0.3, 1.82 ± 1.59, 1.08 ± 1.01, 2.85 ± 4.19, 0.93 ± 1.15 bursts per minute, respectively. However, our informal observations indicated that as early as 2 weeks these network wide bursts of activity would often appear to begin in Well A and after a visible delay, begin in Well B with a latency that was correlated with the number of tunnels connecting each well.

To provide a more robust estimate of this process, short biphasic electrical pulses were applied to an electrode selected among those in Well A, and repeated for an electrode in Well B, that could reliably evoke a burst of activity within that well when stimulated. Each burst evoked by a stimulus in Well A, would sometimes propagate into Well B after a short delay as a second burst of activity in Well B. Examples of this propagation recorded from the 5, 10, 15, and 51 tunnel devices are depicted as raster plots of action potentials recorded on each electrode (vertical axis) in Figures 4A–D. A single stimulus delivered at time 0 (indicated by arrows in Figure 4) evoked a burst of activity across the population of neurons in Well A (upper half of each panel). As this burst of activity evolves, it could potentially recruit enough activity (via axons in the tunnels) among neurons to initiate a second burst of activity within Well B. Changes in the number of tunnels also appeared to affect the time required to propagate from one well to the other. Figures 4A–D depict the post stimulus time histograms (PSTH) of the average response to stimulation for groups 5, 10, 15, and 51 tunnels. Although there is an immediate response in each panel during a stimulus in Well A as indicated by each raster the delay between the response in Well A and the majority of spikes responding in Well B decreases with increasing number of tunnels. This delay is reflected in the PSTHs as a decrease in the delay between peak activities during each burst within each well (indicated by dashed lines).

**Figure 5:**
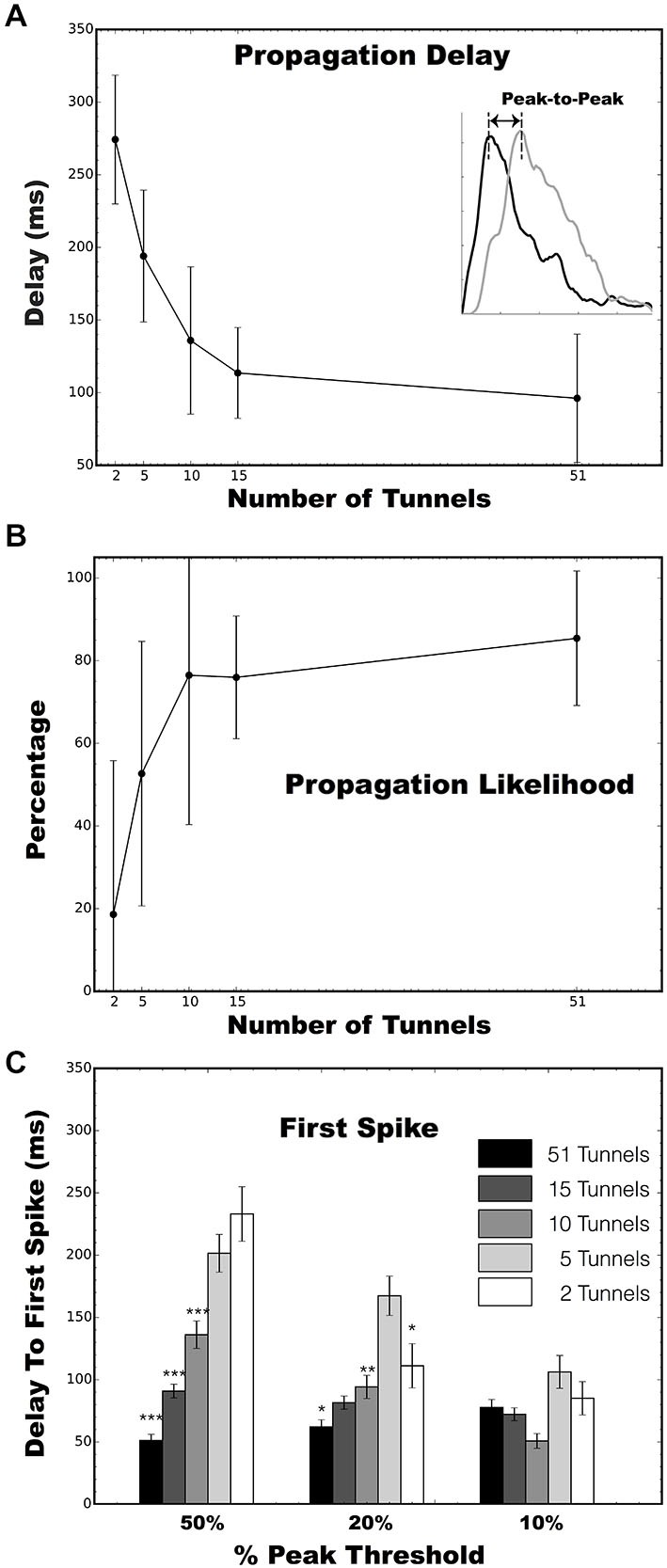
**Analysis of evoked burst propagation between two wells. (A)** Peak to peak latency during propagation of a burst from Well A to Well B as a function of the number of microtunnels. **(B)** Percentages of network wide bursts of neural activity in Well A that successfully propagate to Well B as a function of the number of microtunnels. There were 4, 4, 6, 5, and 4 MEA cultures in the 2, 5, 10, 15, and 51 tunnel groups, respectively. **(C)** Latency to the first spike in Well B following a stimulus in Well A at the 50, 20, and 10% of peak firing thresholds. Error bars indicate the 95% confidence interval. (*p < 0.05, **p 0.01, ***p < 0.001).

We quantified the temporal delay between an evoked burst in Well A and any burst of neural activity that followed in Well B as the difference in the times of peak activity during each burst (illustrated with dashed vertical lines in each PSTH in Figures 4A–D). To propagate from well to well neurons firing during each burst in Well A must recruit enough activity among neurons in Well B to initiate a burst among the population of neurons in Well B. The difference in the timing of peak activity in one Well vs. the other is one objective way to represents the time required for this process of recruitment during successful propagations. That is, it ignores the activity that occurs within Well B that does not lead to a burst event. For example, in Figure 4A a number of spikes occur *before* a full blown burst was able to be evoked in Well B midway through that time window. We hypothesize that more tunnels provides increased communication and stronger connections between populations and that this increase should result in faster recruitment and therefore, shorter propagation delays. Figure 5A plots the average peak-to-peak delay for each group. Increasing the number of tunnels connecting the two wells led to significant decreases in the time delay between bursts from nearly 300 ms in the 2 Tunnel group to less than 100 ms in the 51 Tunnel group (Figure 5A). The probability of each electrically evoked burst in Well A propagating to Well B was also affected by the number of connecting tunnels. We calculated the percentage of trials in which activity evoked in Well A was able to propagate into and produce a burst of activity in Well B (Figure 5B). This percentage is a simple measure of how effective the connections may be at conducting bursts across wells and increased rapidly as the number of tunnels increased from 2 (20%) to 5 (50%) to 10 (80%). This increase in the likelihood of propagation appeared to plateau at 10 tunnels with only small increases beyond that point from 10, to 15, to 51 tunnels. However, even as little as two microtunnels, carrying approximately 20 axons, were occasionally able to propagate a burst between wells (approximately 20% of the time), but did so with significant delays. By comparison, stimulating Well B resulted in fewer trials in which propagation occurred in the reverse direction (0.0%, 12.2 ± 8.3%, 27.6 ± 13.6%, 61.2 ± 20.5%, and 0.0 ± 0% from Well B to Well A for 2, 5, 10, 15 and 51 tunnels). This difference was however, highly variable between individual cultures and likely reflects both the degree to which feed-forward connectivity dominates due to timed plating (Pan et al., 2011, 2014) and impact of reducing the number of tunnels.

Figure 5C plots the average latency between a stimulus in Well A and time to the first spike within each burst measured at thresholds of 10, 20, and 50 of peak firing rates. Like peak-to-peak propagation delays, the time to a first spike following stimulation increased with decreasing number of tunnels connecting each well at 50 and 20% of peak thresholds. There was no significant difference between groups at the 10% threshold due to the increased inclusion of the highly sporadic pre-burst spiking we noted earlier (e.g., Well B; Figure 4A). Mean latencies between A and B decreased with lower thresholds of 70.13 ± 3.55 ms at 10%, 89.29 ± 4.56 ms at 20%, and 112.28 ± 5.43 ms at 50%, *p* < 0.05.

### Functional Connectivity Measures

**Figure 6:**
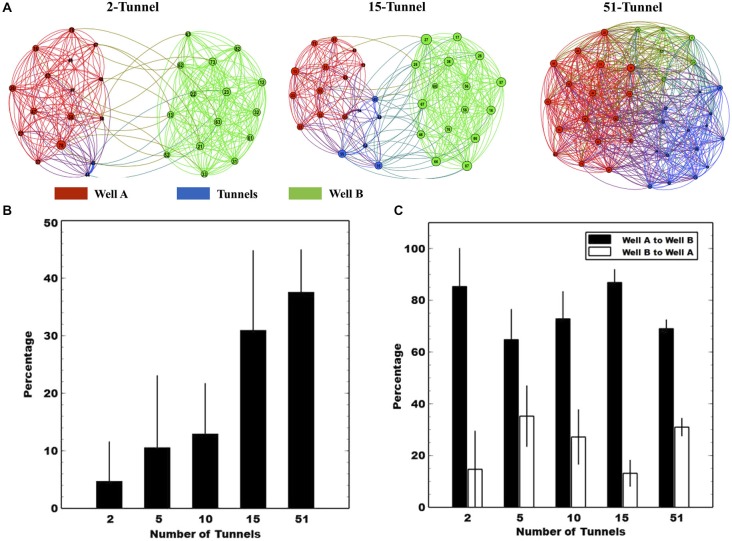
**Conditional granger causality (CGC) analysis. (A)** The functional connectivity reflected by CGC values in three representative subjects of corresponding groups. The nodes denote the electrode and are colored according to the location (Black denotes electrodes in Well A and Gray in Well B). The network is displayed using a force-directed method to show the difference in the functional connectivity between different types of networks (Bastian et al., 2009). **(B)** The percentage of functional connections that originate from neurons in Well A and connect with neurons in Well B relative to all functional connections in Well A. We also assessed the directionality of propagation along the axons located within each tunnel. Panel **(C)** plots mean normalized CGC percentages of forward vs. reverse propagation derived from electrode pairs *located along the tunnels*. Each solid black bar represents the mean CGC estimates in the direction from Well A to Well B while the white bar represents the mean values in the opposite direction. Values from Well A to Well B are higher than those in the opposite direction.

To understand how manipulating the number of tunnels affected the functional connections between the two neural populations, we calculated Conditional Granger Causality (CGC) as a measure of functional connectivity, computed between the neural spike trains from electrode pairs across the MEA. Figure 6A illustrates differences in the pattern of connectivity produced by differing number of tunnels within and between the two populations of neurons. A network graph is plotted from a single culture in the 5, 10, and 51 tunnel groups. Electrodes in Well A, Well B, and tunnels are depicted by the red, green, and blue nodes respectively, and each are numbered by electrode location (column × row relative to the 8 × 8 grid, see Figure 1C). The layout for each network graph was based on a force directed drawing algorithm in which an attractive force is applied between each node pair (Kamada and Kawai, 1989). The force is based on the estimated Granger causal weights. In each group the CGC strengths among nodes within a well draw those nodes together forming clusters for each well. Hence, as the number of tunnels changes any clustering of nodes would represent the competition between the attractive forces within each network and those between networks (i.e., wells). The example networks shown in Figure 6A illustrate the general result of that competition in which nodes appear to cluster by well in all groups but gradually appear to merge as the number of tunnels increase. For example, when 5 tunnels connect the wells, the clusters appear clearly separated, but, when 51 tunnels connect the wells, the clusters coalesce.

To quantify the functional connection strength between Well A and Well B we calculated the percentage of connections from A to B relative the total number of connections in A and B estimated by CGC. The results are plotted in Figure 6A. As the number of tunnels increased the percentage of functional connections between neurons in Well A to those in Well B increased from 5 to almost 40% implying that, with 51 tunnels, neurons in Well A were equally well connected to neurons on the other side of the tunnels in Well B as they were to neurons within Well A itself (*p* < 0.01).

In this study, we also employed timed sequential plating to promote directionality among those connections between Well A and Well B. Figure 6C breaks each bar in Figure 6B down by direction plotting a comparison of the directional bias as a percentage of total connections from Well A to Well B and that from Well B to Well A for each group. In each group, any connections that travel between wells are strongly biased to be from Well A to Well B (*p* < 0.05). This bias is also consistent with an earlier report from our group using time-sequentially plated cultures that showed an 83% directional bias indicated from delays in spike timing of individual action potentials measured as they propagate along the tunnels containing electrodes (Pan et al., 2011).

## Discussion

In this study, we employed custom MEMs devices that differ in the number of tunnels that connect two living cortical networks cultured *in vitro*. By manipulating the geometric connectivity (i.e., number of tunnels) we hypothesized that increasing the number of tunnels would increase the functional strength between these neural populations in Well A and B. We provide three lines of evidence that the number of tunnels did in fact modify the functional strength between each population. First we show that increasing the number of tunnels resulted in an increased likelihood of a burst in Well A to successfully initiate and propagate to Well B (Figure 5A). Second, we show that increasing the number of tunnels also increased the apparent strength of the connection between wells whose strength as reflected by a substantial and significant decrease in peak-to-peak delay (Figure 5B) and first spike (Figure 5C) with which these bursts propagated from A to B. Third, we estimated the functional strength from Well A to Well B vs. Well B to Well A using our functional connectivity measure based on Granger causality and found a robust increase in connectivity as well as a bias in directionality. These effects were made possible by the use of MEMs microtunnel fabrication technology coupled with multi-electrode array and the use of delayed sequential plating of the second neural population in order to create nearly unidirectional “feed-forward” connectivity between populations. We believe that this experimental paradigm can be of use to neuroscientists who utilize cultured neural networks for a variety of studies in which the connectivity including strength, direction, or even location may be manipulated.

To explain our results, we posit a relatively simple hypothesis. By increasing the number of tunnels between the two neural populations we increase the number of connecting axons between each population. Increasing the number of connecting axons should therefore provide greater coupling between the two populations. This in turn should lead to an increase in the likelihood and a decrease in the amount of time that a burst in Well A is able to recruit activity in Well B to then propagate into that well. Burst initiation and propagation is however, a highly complex process. At the micro-level, which neuron(s) are activated during the early period of burst formation can not only determine whether a burst will occur (Maeda et al., 1995; Yvon et al., 2005; Eytan and Marom, 2006; Eckmann et al., 2007, 2008; Cohen et al., 2008; Ham et al., 2008; Pan et al., 2009a,b; Orlandi et al., 2013), but may also determine the pattern of firing that then follows during that burst (Eckmann et al., 2008). *In vivo*, the characteristic timescale of burst activation is on the order of 100–200 ms whether measured in the sensory (Supèr et al., 2001; Slovin et al., 2002), somatosensory (Derdikman et al., 2003), or motor areas (Riehle et al., 1997) or during purely internal events (Riehle et al., 1997). It is also the time required to activate a single cortical column (Derdikman et al., 2003). In this study a similar range of values are observed in our data and parallel reports by other laboratories laboratories using MEA cultures (Eytan and Marom, 2006).

In the Eytan and Marom (2006) study they first showed that select neurons (so called “leader” neurons) preceded each burst, that membership among this select set of neurons was maintained over many hours, and that leader neurons appeared and were predictive of bursts in both spontaneous and evoked activity. They also reinforce the notion that although a burst event appears to be an all-or-nothing threshold-governed event, increasing the number of neurons and hence, action potentials that participate *during* *the initiation* of a burst *decreases* the amount of time needed to reach synchrony (peak-firing) within that burst. This effect may perhaps be comparable to our two-well system in which we increase the number of tunnels and hence, increase the number of “neurons” and activity associated with those neurons that propagates along axons through the tunnels into the opposing well. Perhaps more importantly, Eytan and Marom (2006) also demonstrate that *which neuron from Well A* provides input into Well B may be crucial to determine the delay to bursting in Well B. In a second experiment, Eytan and Marom (2006) electrically coupled two independent cortical cultures, labeled X and Y, using a stimulus generator whose stimulation was timed with spiking on select electrodes in X and stimulus (50 uA, 400 ms bi-polar pulse) delivered to a fixed location in Y (X→Y). Since these cultures are independent there is little reason to believe that the choice of neuron from culture X would influence the delay to a corresponding burst in Y. However, they showed that when those neurons selected to provide input in X were putative “leader” neurons they were far more efficient at eliciting a burst and doing so faster in Y than other neurons among culture X. Their argument was that when culture Y “reads” activity from X through the activity of poorly connected neurons located in X the time delay between bursts in X and Y is longer. However, when a strongly connected neuron among culture X is read by Y, bursts in Y appear almost simultaneously with, or in some cases actually slightly temporally precede bursts in X. In our study we decrease the number of tunnels between each neural population. Perhaps by decreasing the number of tunnels we are also decreasing the likelihood the neural population in Well B has access to stimulation (spiking) by one of these strongly connected neurons in Well A. Information that according to this experiment, would lead to faster time to synchronization (i.e., bursting) relative to Well A. If true, a question might then be how many tunnels are actually needed to improve the synchronization and subsequent propagation of bursts from Well A to Well B?

If we assume one 3 × 10 μm tunnel contains approximately 10 axons, by our estimation 10 microtunnels with approximately 100 total axons would appear to be sufficient to successfully propagate 80% of bursts initiating in Well A into Well B with a relatively small time delay of around 100 ms. Increasing the number of tunnels beyond 10 provides little added benefit, at least in terms of recruiting bursts between wells which appears to plateau at 10–15 tunnels (see Figure 5B). For the given area of each well (20 mm^2^) and a given cell density (approximately 1500 cells/mm^2^) the total number of cells in each well would be 30, 000. Based on these course estimates, only a small fraction of activity (and propagation along axons into the adjacent well) among a population as small as 0.3% (100:30, 000) of the total number of cells in each well would be sufficient to transmit burst activity reliably. Of course, it is likely that not all tunnel axons are simultaneously active, hence the critical number and ratio are likely to be lower, but our estimates provide a crude upper bound on the minimum number of projecting axons needed for a coupling strength high enough, or alternatively provide access to enough activity among “leaders” in Well A, to reliably initiate bursting in the Well B with relatively short delays.

At the macro scale, sensory processing, cognition, and motor control all appear to dynamically engage select neural populations within the brain. During this process activity may remain localized in space and time to a particular area or may propagate as a wave betwee distinct neural populations. Waves are a natural mode of information propagation (Ermentrout and Kleinfeld, 2001; Rubino et al., 2006). These wave fronts are composed of brief bursts of spikes that sweep across the network (e.g., Keane and Gong, 2015; Townsend et al., 2015). One key characteristic of those waves is the speed with which they propagate. *In vivo* and *in vitro* studies indicate both fast (~100 mm/s, e.g., Contreras and Llinas, 2001; Benucci et al., 2007; Xu et al., 2007) and slow propagation speeds (~10 mm/s, e.g., Wu et al., 1999; Sanchez-Vives and McCormick, 2000; Han et al., 2008) in rodent cortex. In cultures in which cells are arranged into a single contiguous line similar propagation speeds ranging from 2–15 mm/s (Feinerman et al., 2005). If we assume a typical well-to-well distance of 1 mm in this study, we find peak-to-peak delays of approximately 100–300 ms. This implies a propagation speed of 3.3–10 mm/s which is remarkably similar to *in vivo* estimates. These velocities are of course, much slower than those associated with conduction along a single axons at 180–1140 mm/s reported by our group (Dworak and Wheeler, 2009; Pan et al., 2011) and others (180 and 930 mm/s, e.g., Colombe and Ulinski, 1999; Kondo et al., 2004). The mechanisms governing wave propagation are however, a matter of ongoing investigation. These mechanisms include cellular and synaptic properties, structural connectivity including distribution of connection lengths composed of combinations of short and long-range connections (i.e., small-world connectivity) (see Wang, 2010 for review) or degree of synaptic connectivity (e.g., Ermentrout, 1998; Golomb and Ermentrout, 1999) that we explicitly attempted to manipulate here. When those mechanisms including structural and oscillatory dynamics break down, the result may lead to a variety of neurophysiological disorders including schizophrenia (e.g., Liu et al., 2008b; Lynall et al., 2010) in which functional *dysconnectivity* is thought to play a role (Stephan et al., 2009; Phillips and Uhlhaas, 2015), autism (Uhlhaas and Singer, 2006; Rippon et al., 2007; Uhlhaas et al., 2009) among other neurological diseases and disorders (He et al., 2007, 2009). In this study, we selectively decrease the number tunnels and hence, coupling between two (and in the future perhaps many more) neural populations. To the extent that this decrease mimics this breakdown or dysconnectivity between areas we believe the methodology developed here may provide a new way with which to study these diseases while at the same improving our understanding of the role of structural connectivity in the propagation of waves, information, and other oscillatory phenomena.

## Conclusion

In this study, we designed and constructed a relatively simple device cast from a mold and made of PDMS that contained differing number of tunnels in an effort to directly manipulate the degree of connectivity between two small populations of cortical neurons. We show that the reliability and latency with which synchronous activity propagates between these networks could be manipulated with as few as 2 tunnels, and would increase in reliability with as few as 10 tunnels and even more so up to the 51 tunnel devices that we tested. With this technology, investigators can now manipulate the strength between two or more populations of neurons to examine the effects of connectivity on the neural population dynamics and transmission of synchronous activity. Perhaps the most important feature of the method we describe here is the unique unidirectional nature of this culture system, which in combination with a designable connectivity (e.g., number of microtunnels or location of those tunnels) will enable investigators to create an array of networks with which to study. Recently, very high-density electrode arrays with over 4,000 electrodes have become commercially available (e.g., Maccione et al., 2010; Timme et al., 2014; Ullo et al., 2014). The combination of these new arrays possessing state-of-the-art spatial resolution over much wider recordings areas than that possible with the arrays used here, coupled to microtunnels whose manipulation (e.g., number, size, location) can determine the functional strength between, will permit a new era in the study of the effects on the dynamics and transmission of these bursts across networks of various topologies at extraordinary levels of detail.

## Conflict of Interest Statement

The authors declare that the research was conducted in the absence of any commercial or financial relationships that could be construed as a potential conflict of interest.
